# Effects from the Freezing of Either Whole or Crushed Grapes on the Volatile Compounds Contents in Muscat Wines

**DOI:** 10.3390/foods11121782

**Published:** 2022-06-17

**Authors:** María del Carmen Pedrosa-López, Fátima Aragón-García, Ana Ruíz-Rodríguez, Zulema Piñeiro, Enrique Durán-Guerrero, Miguel Palma

**Affiliations:** 1 Department of Analytical Chemistry, Center of Agri-Food and Wine Research (IVAGRO), Faculty of Science, University of Cadiz, 11510 Puerto Real, Spain; mariipelopez3@gmail.com (M.d.C.P.-L.); fatima.aragon@uca.es (F.A.-G.); enrique.duranguerrero@uca.es (E.D.-G.); miguel.palma@uca.es (M.P.); 2 IFAPA Rancho de la Merced, Carretera de Trebujena, Km. 2.2, Apdo. 589, 11471 Jerez de la Frontera, Spain; zulema.pineiro@juntadeandalucia.es

**Keywords:** aroma, cryoextraction, prefermentative maceration, Muscat grapes

## Abstract

The transfer of aromatic compounds from the grape skins to the musts has been studied using a process involving freezing whole bunches or crushed grapes for winemaking the Muscat of Alexandria variety (white wine). Subsequently, a prefermentative maceration has been applied to some of the samples. The aromatic profiles of the final wines have been determined using gas chromatography coupled to mass spectrophotometry (GC-MS). The results revealed that, in the trials in which whole grapes were frozen, the final wines had a higher aromatic concentration compared to that of wines obtained by either freezing crushed grapes or obtained with traditional winemaking techniques. Thus, the wines produced from frozen whole grapes were found to exhibit different characteristics from the rest of the wines. The compounds affected by the freezing either of the whole bunches or the crushed grapes were terpenes, acids, and esters. Lower differences were found for wines produced applying prefermentative maceration after the freezing process.

## 1. Introduction

Varietal aroma is an important characteristic in wine quality. Winemaking techniques determine the specific processes to guarantee the compounds related to the varietal aroma are transferred from grapes to final wines. The compounds responsible for this are found in the skin of the berries and, to a lesser extent, in its pulp [1,2]. The Muscat of Alexandria variety is characterized by a high concentration of both bound terpenoids and free aromatic terpenoid compounds. Specifically, the majority of terpenes described in the variety are monoterpene alcohols, including linalool, citronellol, nerol, geraniol, and α-terpineol usually in the ranges of ng L^−1^ to mg L^−1^ and with odor activity values higher than one. They specifically contribute to floral and fruity aromas in wines [3,4]. Other volatile compounds contributing to the aroma of this kind of wines are some norisoprenoids, fatty acids, ethanolic esters, several acetates, and some volatiles phenols, as it occurs with most young white wines [3]. A common technique for winemaking consists in applying a prefermentative maceration process of the grapes’ skin and the must to boost the extraction of the aromatic compounds from the skins [5]. The maceration of red wines, which includes the fermentation stage and even a stage before and/or after fermentation to extract anthocyans, takes a long period of time [6]. On the other hand, the maceration of white wines tends to take a shorter period of time and occurs at lower temperatures so as to avoid the co-extraction of other compounds that could be detrimental to the quality of the final white wine, even producing a very fast browning process [7]. For example, in a study carried out on the white variety Maturana Blanca, a very short prefermentive maceration produced more aromatic wines showing higher contents in ethyl esters and acetates [8]. 

On the other hand, extraction efficiency can be enhanced by combining certain techniques along with skin maceration. Specifically, when working with the Muscat variety, ultrasound applied during a pre-fermentative maceration at 10 °C favors the extraction of compounds and produced more aromatic wines [9]. A freezing process applied over whole berries of Muscat of Alexandria, followed by 3 h skin maceration, produced significantly different wines with increases up to 300% in volatile terpenoids; moreover, higher scores for fruit and flower notes in a tasting panel analysis were reported [10]. Additionally, wines with higher aromatic intensity were obtained with prefermentative maceration (15 °C for 6 and 12 h) in Muscatel de Bornova and Narince varieties [6]. Similar effects were observed in other grape varieties; for example, according to Baron et al., the content of monoterpenes in the Irsai Oliver variety, which is similar to Muscat, doubled the content of linalool and geraniol in the wine after 24 h of maceration at 14 °C [11]. Grapes from the Sauvignon Blanc variety were also used in freezing experiences with later prefermentative maceration processes, specifically using 24 h of maceration after freezing the crushed grapes [12]; the aromatic compounds increased their final concentration. A similar result has been reported for Pinot Blanc grapes [13] after applying a 24 h prefermentative maceration at 4 °C. Prefermentative maceration for 4 days at 6 °C was also effective as esters and higher alcohols content increased in Tempranillo or Tanat varieties [14,15]. However, some additional effects can appear when boosting the extraction process. In the case of Sauvignon Blanc [12], it was observed that the wines produced with prefermentative maceration using dry ice to freeze the crushed grapes showed that the final concentration of phenolic compounds increased between 28% (handpicked grapes) and 11% (machine harvested grapes), with differences in both cases vs. the wines produced without prefermentative maceration. For Pinot Blanc, the major differences were found for phenolic contents in wines produced with or without prefermentative mcaceration at 4 °C for 24 h [13].

Grapes can be frozen using different methods, and the freezing step can be carried out instantly using dry ice or freezing chambers for several hours or even days at −20 °C [10]. The freezing process breaks the grapes’ skin cells and facilitates the transfer of the compounds located in the solid parts into the must when it returns to room temperature [10]. A study on the effects of freezing rates of grapes on the final wine was carried out on the Muscat variety. Thus, the grapes were either instantaneously frozen by means of liquid nitrogen or using a freezing chamber for 40 min. The result was that the instant-frozen wines were more aromatic than the chamber-frozen wines with a higher extraction of terpenes and fatty acids and no negative notes to be mentioned [10]. Another aspect to be pointed out is that the wines that had been elaborated through freezing grapes were found to be more aromatic than the control wine for which its grapes had not been frozen [10]. It has been also reported that freezing the juice or grapes had a cultivar-dependent impact on the free volatile compound profile compared with fresh samples [16]. This winemaking technique was applied to Sauvignon Blanc grapes and resulted in wines with a more complex aroma and a greater polyphenolic load [12]. These effects are explained by the fact that the membranes of the skin cells had been partially or completely broken by the formation of ice crystals during the freezing process, which favored the release of polyphenols and other compounds that confer wine with its varietal aroma, as well as the release of greater amounts of other substances that are present in the skins [10].

Therefore, this study aimed to determine the effects of different freezing procedures during the winemaking processes of Muscat grapes on the content of the main volatile compounds related to the aroma of wine properties. Afterward, the combined specific effects with a prefermentative maceration process will be also evaluated. A specific analysis of the effects on the contents of individual terpenes in the final wines will be performed. Terpenes are the most important compounds in the varietal aroma of several grape varieties, specifically in Muscat grapes, and they are located mainly in grape skins.

## 2. Materials and Methods

### 2.1. Grape Variety and Winemaking Process

Muscat of Alexandria grapes were harvested at Chiclana de la Frontera, Cadiz (lat. 36°25′48.5″ N, lon. 6°06′30.1″ W) when they reached an acidity of 3.6 g L^−1^ of tartaric acid and a sugar level of 23.2 °Brix. Stems were not lignified yet. After harvesting, the grapes were divided into three batches, each of which was subjected to a different winemaking treatment as follows:

Reference treatment (R): Destemmed and traditionally milled, without any freezing process;

Crushed grape freezing (M): The bunches were destemmed, and the grapes were crushed and frozen in a chamber at −18 °C;

Whole bunch freezing (B): The bunches of grapes were frozen directly in a freezing chamber at −18 °C.

In both cases, the freezing period used was 14 days at −18 °C.

**Figure 1:**
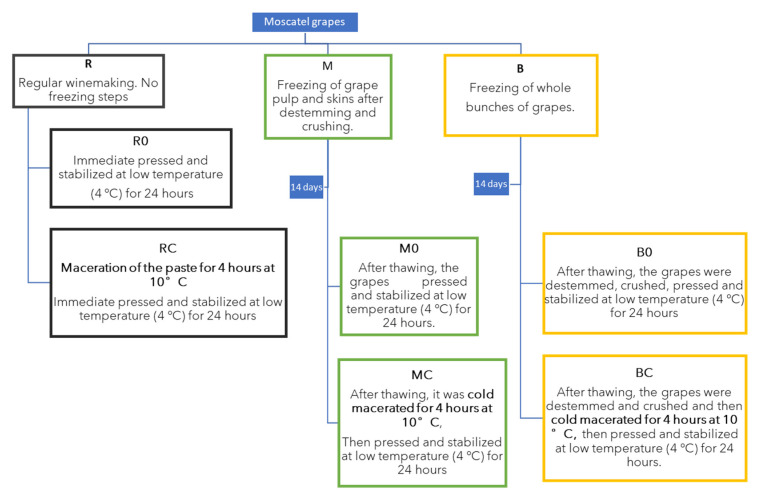
Description of the different winemaking procedures applied to the Muscat of Alexandria grapes. R0: regular winemaking; RC: regular winemaking with prefermentative maceration; M0: frozen crushed grapes then regular winemaking; MC: frozen crushed grapes and prefermentative maceration; B0: frozen bunches then regular winemaking; BC: frozen bunches and prefermentative maceration.

These trials were divided into two sets: For the first set, the wines were vinified after the freezing and/or thawing, and for the second set, a 4 h skin maceration at 10 °C was performed. All trials were carried out in triplicate. Figure 1 describes the different winemaking procedures and the codes used for their identification.

After pressing and cold stabilization, the 18 trials (6 winemaking conditions in triplicate) were treated in the same way: Juice was separated from the solid parts. Steel containers with 50 L capacity (diameter: 30 cm, height: 70 cm) containing approximately 45 L of must were used for the fermentation process. The pH of the must was adjusted using tartaric acid to 3.3 as it is suggested for the specific yeast to be used for alcoholic fermentation. Sulphur dioxide (SO_2_) was added by using potassium metabisulphite to obtain a final concentration of 40 mg L^−1^. 

Active dry yeast *Saccharomyces cerevisiae* var. Bayanus VINIFERM Revelacion (Agrovin, Alcazar de San Juan, Spain) was used for alcoholic fermentation, which was carried out at 16 ± 1 °C in a cold chamber during 10 days. Yeast was prepared following the suggested procedure by the dealer; specifically, a ratio of 100 g of yeast per liter of water at 35 °C was used for its hydration. After 15 min, 0.2 g of yeast per liter of must was added to each fermentation tank. The fermentation was considered completed when the level of the reducing sugars was below 5 g L^−1^.

Finally, the wines were allowed to settle statically for eight days and filtered through cellulose 20 µm pore size plate filters. The wines were stored in 10 L *bags in box* deposits at 15 °C, with the addition of potassium metabisulphite until a total sulphur dioxide concentration of 60 mg L^−1^ was obtained. All analyses were completed between 2 and 3 months after fermentation was completed.

### 2.2. Characterization of the Wines

The must and wine analytical parameters that were monitored over the trials were as follows: sugar level, expressed in °Brix, determined by a DMA 4500 M densimeter (Anton paar GmBH, Graz, Austria), acidity by acid-base titration using a Crison pHmatic 23 (Barcelona, Spain), and the alcohol content, obtained by distillation of the wines and subsequently by determining the density of the distillate using the above-mentioned densimeter, according to the regular methods on wine analyses [17].

### 2.3. Determination of the Aromas

Volatile compounds were determined using the Stir Bar Sorptive Extraction (SBSE) technique and, subsequently, by gas chromatography coupled to mass spectrometry (GC-MS), using an Agilent Technologies 5975 C inert MSD model with Triple-Axis Detector. The determinations were carried out on 6 trials (R0; RC; M0; MC; B0 and BC), using 3 tanks produced under the same conditions, thus reaching a total of 18 wines, which in turn were analyzed in duplicate in order to verify the results of the final samples.

SBSEs were carried out using 10 mm × 0.5 mm (length × film thickness) PDMS commercial stir bars, supplied by Gerstel (Mulheim an der Ruhr, Germany) following the method by Vasile-Simone et al. [18]. Each sample was run in duplicate and the average values of the six types of wine corresponding to each winemaking process were used for later discussions. The Wiley 7N library (Wiley Registry of Mass Spectral Data, 7th Edition, 2000, John Wiley & Sons, Hoboken, NJ, USA) was used for peak identification, and they were confirmed by pattern retention indices, whenever possible, or according to the retention data reported in the literature. The data for the identified compounds were obtained by measuring the relative area of the peak of each compound in the total current ion chromatogram relative to that of 4-methyl-2-pentanol, the internal standard; therefore, semi-quantiative data were used to compare the effects of the winemaking conditions [18]. Relative standard deviations found for the compounds in samples analyzed in triplicate ranged from 5.65% for α- terpineol to 18.14% for ethyl octanoate. 

### 2.4. Statistical Analyses

The differences attributable to the effect of freezing and/or to the cold skin maceration were evaluated using Student’s *t*-test, performed using Microsoft Excel with a confidence interval of 95%.

To determine the differences between the replicates of each trial, cluster analysis was applied using RStudio software (RStudio, Boston, MA, USA).

## 3. Results and Discussion

### 3.1. Characterization of Must and Wines

Table 1 includes the characterization data of the initial must and the final wines along with the results of the Student’s *t*-test; in all cases, the wines produced were compared against the reference wine without freezing procedures, i.e., R0 or RC.

No differences were observed when frozen treatments were compared to the reference must (R0). With regard to the wines’ alcoholic content, it was observed that both the frozen whole grape wine (B0) and the frozen crushed grape one (M0) presented differences in comparison with the reference wine (R0), which is lower. Similar results have been previously reported [19]. In this case, because the sugar levels before the fermentation were not lower, the differences in the final ethanol level could be due to some compounds that were extracted at a higher level, which affected yeast growth.

**Figure 2:**
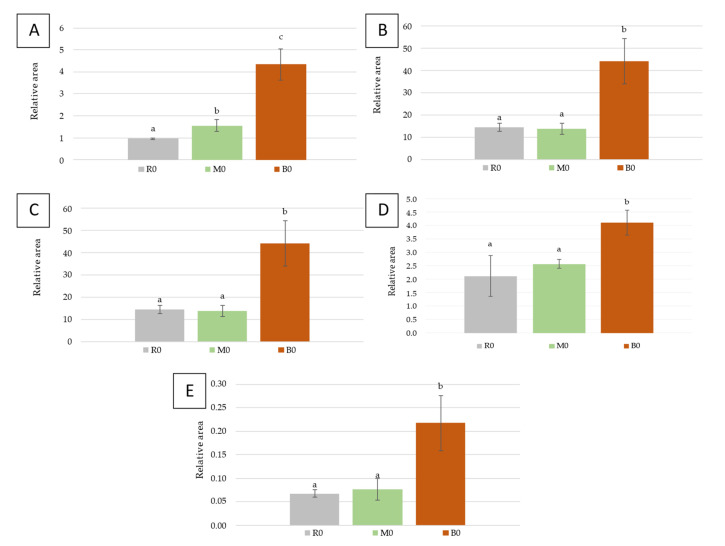
Contents found in wines produced without a prefermentative maceration (*n* = 6). Terpenes (**A**), esters (**B**), acids (**C**), 2-methoxy-4-vinylphenol (**D**), and β-damascenone (**E**). Reference wines (R0); wines from frozen crushed grapes (M0) and wines from frozen whole bunches (B0). Different letters over the bars indicate differences (*p* < 0.05).

The volatile compounds were determined using an SBS-GC-MS equipment that allowed the individualized quantification of a large number and different types of volatile compounds (Table 2), and they are described in Section 2.3. A total of 33 compounds were identified as listed on Table 2 and classified according to their chemical nature:

As a preliminary treatment, the relative area of each compound was calculated based on the internal standard 4-methyl-2-pentanol. Figure 2 shows the results for the contents of terpenes, esters, acids, alcohols, and norisoprenoids in the wines that had undergone a freezing process without prefermentative maceration (R0, M0, and B0).

The effects of the freezing steps during the winemaking process can be clearly demonstrated after reviewing the results of different volatiles compounds. Specifically, a dramatic increase in the content of some volatiles, including the main volatiles related to the specific varietal aroma, i.e., the terpenic alcohols, has been observed. It should be also noted that the prefermentative maceration after the freezing process was not needed to guarantee higher content of volatiles, but even lower values were found. Therefore, freezing procedures of either whole grape bunches or crushed grapes produced much more aromatic wines than the regular winemaking process. Even the very common prefermentative maceration could be replaced by some freezing steps producing higher levels of volatile compounds related to varietal aroma. 

### 3.2. Effects from the Application Freezing Processes without Prefermentative Maceration

After determining 18 different wine samples, the data were grouped according to the family of compounds to facilitate the discussion. 

Terpenes are the most characteristic compounds in the Muscat of Alexandria variety, and they are responsible for the floral notes of its wines [20]. As observed in Figure 2A, the reference wine (R0) registered values of 0.96 ± 0.04 while the frozen crushed grape wine (M0) reached 1.55 ± 0.26 and the frozen whole grape wine (B0) went up to 4.34 ± 0.70.

The effects on the content of terpenes demonstrated the efficiency of the freezing process. Although the frozen crushed grape wine (M0) increased the release of compounds into the medium, the effect caused by freezing whole grapes (B0) significantly enhanced the extraction of the compounds located in the skins by up to 350% compared to the non-freezing process (Figure 2A). In previous works, increases in the concentration of some compounds have been reported when applying cryoextraction techniques; however, they were not as high as those registered in this case [6,10]. Therefore, grape freezing techniques seem to positively favor the varietal character of the grape and, therefore, appear to have a positive effect on the wines.

Figure 2B presents the results for esters, showing clearly how the wine from frozen whole grapes (B0) achieved much higher values than the reference wine, 44.14 ± 10.13 versus 14.57 ± 1.85, which represents an increase higher than 200%. Esters are the substances formed by the combination of an organic acid and alcohol. Many of them, namely ethyl esters, originated during the wine fermentation process when ethanol appears to form esters with the fatty acids present in the wine [21]; thus, their increase is related to a higher extraction of fatty acids, and they are associated with floral and fruity aromas in young wines [20]. As with the terpenes, the contents of esters for wines produced using frozen whole grapes (B0) were much higher than the other wines: either R0 or M0 (Figure 2B). No differences were found between R0 and M0. It should be noted that freezing treatments are expected to damage the cells; then, a larger extraction of compounds from the solid parts of the grapes is permitted. In this case, after freezing the crushed grapes, there were no higher values for esters. It can be thought that further extraction of various compounds may produce some degradation reactions of some fatty acids and then lower values were produced for the resulting esters.

Figure 2C illustrates how, in the acid group, the frozen whole bunch of grape trials (B0) exhibited differences with respect to the reference wine (R0), and it was also different from M0. In fact, B0 reached values of 11.19 ± 2.38, which are almost three times higher than those of the reference wine (4.07 ± 1.00). These results were also similar to the results on terpenes and esters. The wine produced using frozen whole grapes showed higher content for acids. On the other hand, the wines made by freezing crushed grapes did not show a clear increase with respect to the non-frozen reference wine. The fermentation conditions and the treatment applied to the grapes may affect the fatty acid composition of the wines [7]. In this study, the fermentation conditions were the same for all trials. Therefore, freezing whole bunches allow fatty acids to be easily released from the grape skins; however, the same results were not produced if the crushed grapes are frozen.

Figure 2D shows the results for the phenol (2-methoxy-4-vinylphenol). Wine B0 reached values of 4.11 ± 0.49 compared to 2.11 ± 0.75 obtained by R0; i.e., with practically double amount of this compound, this means freezing whole bunches of grapes would have a negative effect on the wine compared to unfrozen winemaking, since it contributed higher levels of undesirable smoky aroma for young white wines [20]. The frozen crushed grape wine (M0) did not present differences with respect to the reference wine (R0) instead. Therefore, specifically frozen whole bunches of grapes would have a negative effect on the wine compared to unfrozen winemaking, since it contributed higher contents of some undesirable aromas [21,22].

The contents found for β-damascenone, a norisoprenoid, were much higher for B0 wines than for R0 and M0, with no differences between them (Figure 2E). It can be observed that B0 reached three-times larger values than R0 (0.07 ± 0.01) and M0 (0.07 ± 0.02). Therefore, similarly as terpenes and esters, only the wines from (B0) showed differences with respect to the reference wines. This implies that the frozen whole grape wine achieved higher levels of volatile compounds from the grape skins than for frozen crushed grape wine.

### 3.3. Effects from the Application of a 4 h Prefermentative Cold Maceration

Prefermentative maceration usually increases the extraction of compounds from grape skins [21]. This effect could be higher if the skins were previously degraded, which occurs during the freezing process [10]. For that reason, the effects of applying a 4 h prefermentative cold maceration were evaluated by comparing their levels in the final wines.

**Figure 3:**
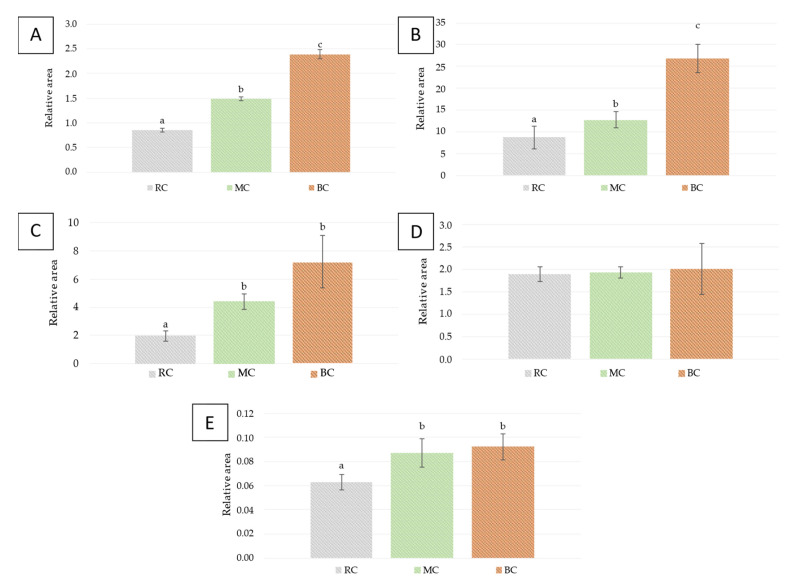
Contents found in wines produced with a prefermentative maceration (*n* = 6). Terpenes (**A**), esters (**B**), acids (**C**), 2-methoxy-4-vinylphenol (**D**), and β- damascenone (**E**). Reference wines (RC); wines from frozen crushed grapes (MC) and wines from frozen whole bunches (BC). Different letters over the bars indicate differences (*p* < 0.05).

In Figure 3A, the relative areas corresponding to the terpenes group in wines produced using a 4 h cold prefermentative maceration can be observed. The MC wines presented differences with respect to the RC ones, reaching values of 1.48 ± 0.08 versus 0.85 ± 0.04, which represents a 75% increment. In the case of BC wines, an increase of 181% was observed with respect to RC wines. Therefore, the wines produced with freezing whole bunches (BC) almost contain double amount of terpenes than the regular wines (RC). Similar effects have been previously reported [10]. It should be noted that terpene contents for wines produced without prefermentative maceration and frozen bunches were 4.34 ± 0.70 (Figure 2A); however, the wines produced with prefermentative maceration reached only 2.39 ± 0.16 (Figure 3A). Therefore around 50% of terpenes extracted due to the freezing process of bunches were lost during prefermentative maceration. This means that the advantages of the freezing process were dramatically reduced due to the application of prefermentative maceration. It could the due to degradation reactions because of some compounds also with higher levels than in the regular winemaking conditions because the reduction found for terpenes was higher in the processes including freezing steps. Even with the dramatic reduction in terpenes, wines produced with freezing steps showed higher contents of terpenes than the wines produced using the regular winemaking process, including prefermentative maceration. 

For the group of esters studied (Figure 3B), the wines produced by skin maceration and freezing of the crushed grapes (MC) showed differences with respect to RC wines, with increases of 46%. Wines made with BC also showed differences with both RC and MC wines. However, that difference was lower than for wines produced without prefermentative maceration, because the contents for esters for BC wines were much lower than for B0 wines (relative areas of 26.84 ± 8.12 for BC vs. 44.14 ± 10.13 for B0).

Figure 3C shows the results for fatty acids. The wines produced after freezing the crushing grapes and whole bunches followed by skin maceration (MC) show higher contents with respect to the RC wines, with fatty acid values reaching 4.42 ± 0.54, which represents an increase of 125%. No differences were found between the two wines produced with a freezing step. If the amounts for wines with and without prefermentative maceration are compared, it can be observed that both reference and wines produced with frozen bunches show a dramatic reduction in fatty acids.

Wines produced with prefermentative maceration did not show differences for 2-methoxy-4-vinylphenol found in the wines (Figure 3D), and similar contents were found for RC, MC, and BC wines. This means that the results found for 2-methoxy-4-vinylphenol were different from those for the previous volatiles. Contents were similar for the three wines. In this case, we can conclude that neither freezing of the berries nor of the crushed grapes had any effect on its content of the wines as long as a skin maceration was performed. In wines produced without prefermentative maceration, higher 2-methoxy-4-vinylphenol contents were found for the wines produced using freezing process for the bunches (relative area for B0: 4.11 ± 0.49 vs. BC: 2.01 ± 0.56); therefore, during the 4 h prefermentative maceration, around 50% of 2-methoxy-4-vinylphenol were either degraded or lost. Therefore, maceration after any freezing process reduces the differences due to the freezing process (Figure 2D).

Regarding the contents of the norisoprenoid (β-damascenone), MC and BC wines showed differences vs. RC wines, as it can be observed in Figure 3E. The values achieved by both elaborations were very similar (0.086 ± 0.011 for MC and 0.092 ± 0.012 for BC), which represents an approximate increment of 30% with respect to RC wines. Wines produced by freezing the whole bunches of grapes did not show higher values of β-damacenone after prefermentative maceration; however, it was a consequence of the dramatic reduction in the contents of wines produced after freezing whole bunches; i.e., they moved from relative areas of 0.216 ± 0.028 without prefermentative maceration (B0) to 0.092 ± 0.012 with prefermentative maceration (BC). Therefore, as it was observed for the terpenes and esters, prefermentative maceration did not increase the values for volatiles, but it produced a dramatic reduction in some of them. If the decrease in these compounds had been produced due to degradation reactions by prefermentative conditions, similar reductions would have been found for the three winemaking conditions. However, MC and BC wines showed more intense reductions in these compounds than RC vs. wines without prefermentative maceration. Therefore, some reactions should be produced in the prefermenative maceration between terpenes, esters, or the norisoprenoid, and some compounds were found in higher levels in MC and BC juices. This result conditions the most convenient post-freezing treatment during the winemaking process; in this case, a prefermentative maceration would not be the most interesting practice. 

### 3.4. Specific Effects on the Levels of Terpenes

From the groups of compounds analyzed, the terpenes presented the most interesting results and were the most characteristic compounds in Muscatel wines, inalo previously reported. Based on the values of the individual terpenes detected (camphene, geraniol, hotrienol, limonene, inalool, nerol, α-terpinene, α-terpineol, α-terpinolene, and β-citronellol), cluster analysis was performed to determine the similarities between the wines produced and to determine the combined effects from the two processes: freezing and cold prefermentative maceration. The Euclidean distance was used for distance metrics, and the total linkage was used for clustering. After an initial review, samples BC1-1, M01-2, and M02-2 were discarded since they presented individual values of some terpenes considered as outliers, i.e., values with a relative difference of more than 50% with respect to the average in each type of wine.

**Figure 4:**
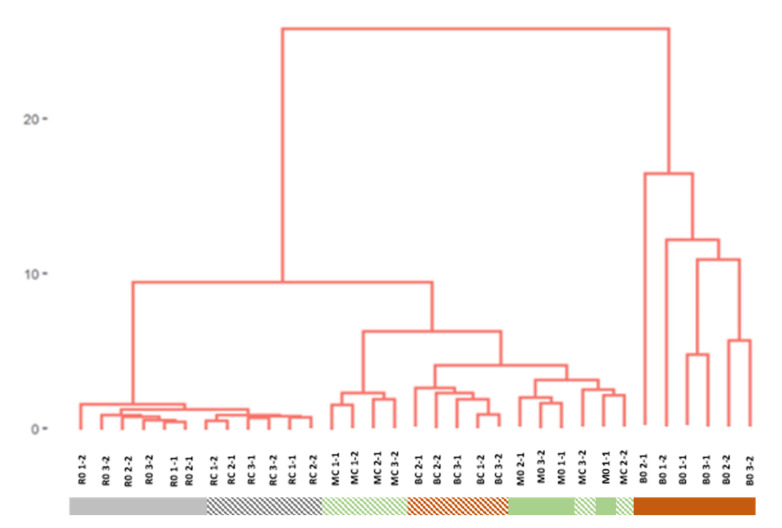
Results from the cluster analysis of the 33 samples from the 18 wines produced through different winemaking procedures. The individual values of monoterpene alcohols were used as variables. Codes used for wines: R: Regular winemaking process with no freezing step. M: Winemaking process with a freezing step of grape pulp and skins after destemming and crushing. B: Winemaking process with a freezing step of whole bunches of grapes. 0: Winemaking without prefermentative cold maceration. C: Winemaking with prefermentative cold maceration.

Figure 4 shows the 33 duplicates analyzed from the 18 wines, where two groups can be clearly differentiated: the first one is a group with a certain heterogeneity but is completely separated from the rest, formed by all the samples from the wines where the bunches of grapes had been frozen and no skin maceration had been performed (B0). The second group includes the remaining 27 samples, i.e., the 12 reference wine samples (R0 and RC), the 10 frozen crushed grape wine samples (M0 and MC), and the 5 wine samples made by freezing whole grapes and also applying prefermentative maceration (BC).

Wines produced freezing whole bunches and prefermentative maceration (BC) do not appear as different as B0. This means that if cold prefermentative maceration is applied after freezing the bunches, the resulting wines have no peculiarity and present similar terpenic contents as the rest of the wines elaborated with or without cold prefermentative maceration (a specific cluster for BC wines in the middle of M0 and MC wines). If we examine more closely the distribution on the graph of the remaining wines, we can differentiate a first subgroup formed by the reference wines, which present a rather uniform distribution. The differences between them because of the application or absence of cold prefermentative maceration are minimal. However, the second subgroup branches out and generates three additional subgroups that correspond to M0, MC, and BC wines. No clear differentiation could be established between the two types of wines (skin-macerated or non-macerated) obtained after freezing the crushed grapes.

In summary, regarding terpene levels, a clear difference can be observed between wines produced with or without the freezing step. Moreover, when whole grapes are frozen and no skin maceration is performed, the wines produced are completely different from the rest, whereas skin maceration makes the wines obtained from different types of freezing more similar.

## 4. Conclusions

In general terms and on the basis of the compounds studied in this work, we can conclude that when freezing is included in winemaking procedures, greater amounts of volatile compounds were extracted into the medium. The freezing of whole berries, in particular, when compared against the freezing of the crushed grapes stands out with increments greater that 100% for the volatiles determined in wines examined in the present study. Some differences were found for different chemical families; however, the most directly related to the floral/varietal notes of Muscat wines, i.e., terpenic alcohols, showed a dramatic increase after freezing either crushed or whole grapes.

The main effect of an additional prefermentative maceration was the smaller differences between the wines made with frozen whole grapes or with frozen crushed grapes, even if they both clearly differentiated from non-frozen wines in most parameters. It can be concluded that prefermentative maceration would not be convenient after any freezing step, as it would produce a reduction in the benefits of freezing either whole grapes or crushed grapes.

Clearly distinguishable contents of the monoterpenic alcohols were found for the wines produced after freezing the whole brunches of grapes, making this technique specifically interesting for winemakers. It must be noted that there will be high costs for the freezing step and large facilities will be needed. However, the time for the grapes to be frozen will allow winemakers to determine a better schedule for winemaking procedures. 

## Figures and Tables

**Table 1:** Main parameters of the starting must and final wines (*n* = 3).

		°Brix	pH	Total Acidity (g L^−1^ Tartaric Acid)			Ethanol (%)
**Must**	R0	22.4 ± 0.07	3.63 ± 0.06	4.67 ± 0.09	**Wine**	R0	12.89 ± 0.05
	RC	22.4 ± 0.07	3.63 ± 0.06	4.69 ± 0.06		RC	13.02 ± 0.02
	M0	23.4 ± 0.05	3.67 ± 0.10	4.15 ± 0.07		M0	12.76 ± 0.03 *
	MC	23.4 ± 0.07	3.68 ± 0.09	4.15 ± 0.05		MC	13.13 ± 0.05
	B0	23.4 ± 0.05	3.87 ± 0.12	4.02 ± 0.07		B0	12.61 ± 0.03 *
	BC	23.4 ± 0.06	3.89 ± 0.11	3.98 ±0.06		BC	13.01 ± 0.04

R: Regular winemaking process with no freezing step. M: Winemaking process with a freezing step of grape pulp and skins after destemming and crushing. B: Winemaking process with a freezing step of whole bunches of grapes. 0: Winemaking without prefermentative cold maceration. C: Winemaking with prefermentative cold maceration. * An asterisk indicates difference (*p* < 0.05) with respect to the control wine without freezing processes (R0 or RC).

**Table 2:** Volatile compounds found in the final wines. The compounds were identified by their linear retention index or/and their mass spectra in SBSE-GC-MS analyses.

	Compound	LRI *	Identification **
Fatty acids	Acetic acid	1428	MS, S, LRI
	Hexanoic acid	1822	MS, S, LRI
	Octanoic acid	2000	MS, S, LRI
	Dodecanoic acid	2160	MS, LRI
	Geranic acid	2203	MS, LRI
	Tridecanoic acid	2239	MS, LRI
Higher alcohols	Isoamyl alcohol	1121	MS, S, LRI
	Phenylethyl alcohol	1883	MS, S, LRI
Esters	Isobutyl acetate	802	MS, S, LRI
	Ethyl butyrate	845	MS, S, LRI
	Isoamyl acetate	989	MS, S, LRI
	Ethyl hexanoate	1155	MS, S, LRI
	Methyl octanoate	1375	MS, S, LRI
	Ethyl octanoate	1441	MS, S, LRI
	Geranyl acetate	1519	MS, LRI
	Ethyl decanoate	1654	MS, S, LRI
	Citronellyl acetate	1672	MS, LRI
	Diethyl succinate	1677	MS, S, LRI
	Neryl acetate	1732	MS, LRI
volatile phenol	2-methoxy-4-vinylphenol	2100	MS, S, LRI
Norisoprenoid	β- damascenone	1820	MS, S, LRI
Terpenes from the alcoholic fermentation	Nerol oxide	1474	MS, LRI
	α- terpinene	1506	MS, S, LRI
	α- terpinolene	1534	MS, S, LRI
	Camphene	1706	MS, LRI
Terpenes from grapes	Linalol	1546	MS, S, LRI
	Hotrienol	1607	MS, LRI
	α- terpineol	1711	MS, S, LRI
	Limonene	1759	MS, S, LRI
	β- citronellol	1764	MS, S, LRI
	Nerol	1793	MS, S, LRI
	Geraniol	1831	MS, S, LRI

* Linear retention indices calculated in a DB-Wax column for the identified compounds. ** Identification by means of standard compounds (S), mass spectra (MS), and/or linear retention indices (LRI).

## Data Availability

All data are included in the manuscript.

## References

[1] Hidalgo Férnandez-Cano L., Hidalgo Togores J. (2011). Tratado de Viticultura.

[2] Hidalgo Togores J. (2006). La Calidad del Vino Desde el Viñedo.

[3] Ebeler S.E. (2001). Analytical chemistry: Unlocking the secrets of wine flavor. Food Rev. Int..

[4] Black C.A., Parker M., Siebert T.E., Capone D.L., Francis I.L. (2015). Terpenoids and their role in wine flavour: Recent advances. Aust. J. Grape Wine Res..

[5] Blesic M., Zele M., Bavcar D., Spaho N., Smajic-Murtic M. (2016). Monoterpenes in cv. Zilavka free-run musts from prefermentatively macerated pomace. Am. J. Enol. Vitic..

[6] Selli S., Cabaroglu T., Canbas A., Erten H., Nurgel C. (2003). Effect of skin contact on the aroma composition of the musts of *Vitis vinifera* L. cv. Muscat of Bornova and Narince grown in Turkey. Food Chem..

[7] Castro Vázquez L., Pérez-Coello M.S., Cabezudo M.D. (2002). Effects of enzyme treatment and skin extraction on varietal volatiles in Spanish wines made from Chardonnay, Muscat, Airén, and Macabeo grapes. Anal. Chim. Acta.

[8] Naranjo A., Martínez-Lapuente L., Ayestarán B., Guadalupe Z., Pérez I., Canals C., Adell E. (2021). Aromatic and sensory characterization of maturana blanca wines made with different technologies. Beverages.

[9] Aragón-García F., Ruíz-Rodríguez A., Palma M. (2021). Changes in the aromatic compounds content in the muscat wines as a result of the application of ultrasound during pre-fermentative maceration. Foods.

[10] Ruiz-Rodríguez A., Durán-Guerrero E., Natera R., Palma M., Barroso C.G. (2020). Influence of two different cryoextraction procedures on the quality of wine produced from muscat grapes. Foods.

[11] Baron M., Sochor J., Prusova B., Tomaskova L., Kumsta M. (2017). A study on the content of terpenic compounds in the cultivar “moravian Muscat” (*Vitis vinifera* L.). Ital. J. Food Sci..

[12] Olejar K.J., Fedrizzi B., Kilmartin P.A. (2015). Influence of harvesting technique and maceration process on aroma and phenolic attributes of Sauvignon blanc wine. Food Chem..

[13] de Matos A.D., Longo E., Chiotti D., Pedri U., Eisenstecken D., Sanoll C., Robatscher P., Boselli E. (2020). Pinot blanc: Impact of the winemaking variables on the evolution of the phenolic, volatile and sensory profiles. Foods.

[14] Aleixandre-Tudó J.L., Álvarez I., Lizama V., Nieuwoudt H., García M.J., Aleixandre J.L., du Toit W.J. (2016). Modelling phenolic and volatile composition to characterize the effects of pre-fermentative cold soaking in Tempranillo wines. LWT Food Sci. Technol..

[15] González-Neves G., Favre G., Gil G., Ferrer M., Charamelo D. (2015). Effect of cold pre-fermentative maceration on the color and composition of young red wines cv. Tannat. J. Food Sci. Technol..

[16] Ouellet É., Pedneault K. (2016). Impact of frozen storage on the free volatile compound profile of grape berries. Am. J. Enol. Vitic..

[17] OIV (2021). Compendium of International Methods of Wine and Must Analysis International Organisation of Vine and Wine.

[18] Vasile-Simone G., Castro R., Natera R., Masino F., Barroso C.G., Durán-Guerrero E. (2017). Application of a stir bar sorptive extraction method for the determination of volatile compounds in different grape varieties. J. Sci. Food Agric..

[19] Schmid F., Jiranek V. (2011). Use of fresh versus frozen or blast-frozen grapes for small-scale fermentation. Int. J. Wine Res..

[20] Niu Y., Wang P., Xiao Z., Zhu J., Sun X., Wang R. (2019). Evaluation of the perceptual interaction among ester aroma compounds in cherry wines by GC–MS, GC–O, odor threshold and sensory analysis: An insight at the molecular level. Food Chem..

[21] Sánchez Palomo E., Pérez-Coello M.S., Díaz-Maroto M.C., González Viñas M.A., Cabezudo M.D. (2006). Contribution of free and glycosidically-bound volatile compounds to the aroma of muscat “a petit grains” wines and effect of skin contact. Food Chem..

[22] Ristic R., Osidacz P., Pinchbeck K.A., Hayasaka Y., Fudge A.L., Wilkinson K.L. (2011). The effect of winemaking techniques on the intensity of smoke taint in wine. Aust. J. Grape Wine Res..

